# Gestational weight gain in Chinese women -- results from a retrospective cohort in Changsha, China

**DOI:** 10.1186/s12884-018-1833-y

**Published:** 2018-05-29

**Authors:** Xin Huang, Hongzhuan Tan, Ming Cai, Ting Shi, Chunmei Mi, Jun Lei

**Affiliations:** 1 Department of Preventive Medicine, School of Medicine, Hunan Normal University, Changsha, Hunan China; 2 Department of Epidemiology and Health Statistics, Xiangya School of Public Health, Central South University, Changsha, Hunan China; 3 Maternity and Child Care Hospital of Yuelu District, Changsha, Hunan China; 4 Maternity and Child Care Hospital of Tianxin District, Changsha, Hunan China; 5 Department of Obstetrics and Gynecology, The Third Xiangya Hospital of Central South University, 138 Tongzipo Road, Yuelu District, Changsha, 410013 Hunan, Province China

**Keywords:** Body mass index, Gestational weight gain, Chinese women, Pregnancy

## Abstract

**Background:**

The generalizability of the gestational weight gain (GWG) ranges recommended by the Institute of Medicine (IOM) to Chinese women is disputed.

**Methods:**

In 2016, 16,780 pregnant women who gave birth to live singletons in Changsha, China, were enrolled. First, subjects with optimal pregnancy outcomes were identified for the GWG percentile distribution description and for comparison to the IOM recommendations. Second, all subjects with optimal GWG according to the IOM body mass index (BMI) cutoffs and those with optimal GWG according to the Asian BMI cutoffs were selected. Pregnancy outcomes were compared between those two groups.

**Results:**

A total of 13,717 births with optimal pregnancy outcomes were selected to describe the GWG distribution. The height and central position of the GWG distributions determined by the Asian BMI cutoffs differed from those determined by the IOM BMI cutoffs among the overweight and obese groups. The recommended IOM GWG ranges were narrower than and shifted to the left of the observed distributions. In both BMI classification schemes, however, the IOM-recommended ranges were within the middle 70% (Pc 15th–85th) and 50% (Pc 25th–75th) of the observed distribution. A total of 6438 (38.37%) and 6110 (36.41%) women gained optimal GWG, according to the IOM and Asian BMI classifications, respectively. Compared with those with optimal GWG according to IOM BMI cutoffs, women with optimal GWG according to the Asian BMI cutoffs had lower risks of both macrosomia (adjusted OR = 0.79, 95%CI: 0.67–0.94) and large-for-gestational age (adjusted OR = 0.86, 95%CI: 0.76, 0.98). However, no significantly different risks of preterm, low birthweight, small-for-gestational age, pregnancy-induced hypertension, or gestational diabetes were found between them.

**Conclusions:**

The IOM-recommended GWG ranges are within the middle 70% of the distributions in Chinese women, and pre-pregnancy weight status should be determined by the Asian BMI cut-off points for monitoring and making GWG recommendations to Chinese women.

**Electronic supplementary material:**

The online version of this article (10.1186/s12884-018-1833-y) contains supplementary material, which is available to authorized users.

## Background

A nutritious diet during pregnancy maintains maternal energy requirements, provides a substrate for the development of new fetal tissues, and builds energy reserves for postpartum lactation [1]. Therefore, the importance of nutrition in pregnancy cannot be overemphasized. Previous studies have proved that excessive or inadequate weight gain during pregnancy has negative implications on pregnancy outcomes, putting the health of both mother and infant at risk [2–4].

To avoid maternal and infant adverse outcomes, in 2009, the Institute of Medicine (IOM), USA, published revised recommended gestational weight gain (GWG) [5] and the revisions was based on pre-pregnancy weight status. Since the effect of weight gain during pregnancy on fetal growth has found to be varied by maternal pre-pregnancy body mass index (BMI) [6, 7] In Jan 2018, the national prenatal care guideline for Chinese women was officially published, but it just duplicated the IOM recommendations for GWG monitoring among Chinese [8]. Those recommendations, however, were made based on Western populations, and their generalizability to Chinese women is under dispute [9–13].

One of the reasons for the conflict may be that BMI cut-off points used in previous studies are inconsistent: Yang et al. [9] used IOM-recommended BMI categories and concluded that IOM GWG recommendations are suitable for Chinese; Zhou et al. [10] and Wong et al. [11] used the Asian BMI cut-off points and found that the IOM GWG recommendations are inappropriate. The weight gain recommendations of the IOM are based on Western BMI cut-off points [5]. However, the Asian BMI cut-off points for determining overweight and obesity in Asian populations are different [13] because researchers found that Asian populations have different associations between BMI and health risks than do Western populations [14, 15]. Under different BMI cut-off points related to different recommended weight gain ranges [5], some Chinese women would be classified in lower pre-pregnancy BMI categories and assigned to larger target weight gain ranges when the IOM BMI cut-off points were used instead of the Asian BMI cut-off points. The appropriateness of using the IOM GWG recommendations for Chinese women should be examined by describing the distributions of GWG by pre-pregnant weight status, as determined using both Asia and IOM BMI cut-off points.

We therefore conducted a retrospective pregnancy cohort study in Changsha, China, 1) to describe the GWG distributions by BMI group, as determined using the IOM and Asian BMI cut-off points, and compare those distributions to the IOM GWG recommended ranges among women with optimal birth outcomes and 2) to compare the pregnancy outcomes among those with the optimal GWG determined using the Asian BMI cut-off points to those with the optimal GWG determined using the IOM BMI cut-off points.

## Methods

### Study population

Our population-based retrospective cohort study was conducted in Changsha city in Hunan Province. Changsha is a city with 6 urban districts (Yuelu, Tianxin, Kaifu, Yuhua, Furong, and Wangcheng district), 1 county (Changsha county) and 2 county-level cities (Ningxiang and Liuyang city). In 2016, a total of 116,336 pregnant women gave birth in Changsha. All pregnant women aged 18 years or older (18,843) who resided in Yuelu or Tianxin district and had a live birth from Jan to Dec 2016 were enrolled when they came to local maternity care units to apply for birth certificates. The sponsors of the study had no role in study design, data collection, data analysis, data interpretation, or writing the report. The study protocol was reviewed and approved by the Ethical and Confidentiality Committee of Central-South University and by both institutional review boards (IRB) from the Maternity and Child Care Hospital of Yuelu District and Tianxin District. All participants provided signed written consent. Women who had multiple births and/or had infants with birth defects, or who had chronic hypertension, diabetes, renal disease or cardiovascular diseases before pregnancy, or were lacking data on GWG were excluded, which yielded a final eligible analytical sample size of 16,780.

### Data collection and variable definition

All data used in the present study were extracted from two sources: antenatal care booklets and hospital discharge abstracts (including both maternal obstetrical delivery records and newborn hospital records). In China, medical information on antenatal care is routinely recorded by certified doctors or nurses in an antenatal care booklet beginning with the first prenatal visit. The booklet is kept by the individual during pregnancy and must be returned to the local maternity care unit, along with the hospital discharge abstract, before applying for an official birth certificate at the hospital. General information on maternal demographic characteristics, pre-pregnancy weight and obstetric history were reported by the interviewee themselves, which could be obtained from antenatal care booklets. Information on weight at delivery, maternal complications and neonatal outcomes was extracted from discharge abstracts.

Pre-pregnancy BMI was calculated as maternal pre-pregnancy weight in kilograms divided by squared height in meters. GWG was calculated by subtracting pre-pregnancy weight from maternal weight at delivery in kilograms. The optimal GWG was defined as the weight gain during pregnancy within the IOM recommended range by pre-pregnancy BMI category. Pre-pregnancy weight status was categorized using both the Asian and IOM cut-off points (Table 1).

**Table 1:** IOM weight gain recommendations for pregnancy by pre-pregnancy weight status

Weight category	IOM BMI category criteria	Asian BMI category criteria	IOM-recommended weight gain
Underweight	< 18.5 kg/m^2^	< 18.5 kg/m^2^	12.5–18 kg
Normal weight	18.5–24.9 kg/m^2^	18.5–22.9 kg/m^2^	11.5–16 kg
Overweight	25.0–29.9 kg/m^2^	23–24.9 kg/m^2^	7–11.5 kg
Obese	≥30 kg/m^2^	≥25 kg/m^2^	5–9 kg

Adverse pregnancy outcomes considered in this study included preterm (delivered at less than 37 weeks of gestation), low birthweight (LBW, birthweight < 2500 g), macrosomia (birthweight ≥4000 g), large-for-gestational age (LGA, birthweight >90th for gestational age and sex), small-for-gestational age (SGA, birthweight <10th percentile for gestational age and sex), pregnancy-induced hypertension (PIH) and gestational diabetes mellitus (GDM). For infants born between 28 and 44 weeks of gestation, birthweight reference percentiles for Chinese infants [16] were used to define SGA and LGA. For infants born between 22 and 27 weeks of gestation, a United States national reference was applied [17]. PIH included gestational hypertension and preeclampsia which was defined as systolic BP (SBP) ≥140 mmHg and/or diastolic BP (DBP) ≥90 mmHg, occurring for the first time after 20 weeks of gestation, with or without proteinuria. Oral glucose tolerance test (OGTT) was routinely examined at 24–28 gestational weeks, and GDM was defined as diabetes that was first diagnosed during pregnancy, with 3-h 100 g OGTT results exceeding cut-offs for two or more values: fasting plasma glucose ≥5.3 mmol/L, 1-h ≥ 10.0 mmol/ L, 2-h ≥ 8.6 mmol/ L and 3-h ≥ 7.8 mmol/ L).

### Statistical analysis

**Fig. 1:**
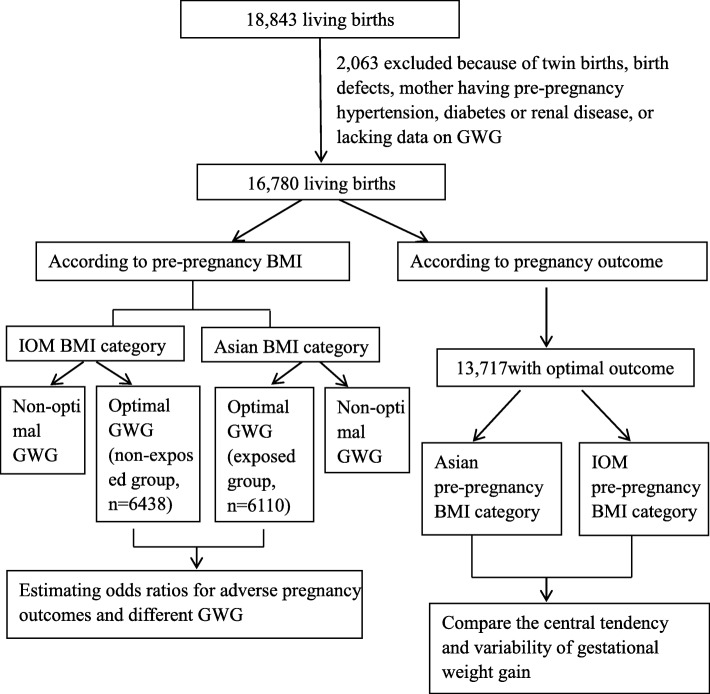
Analytical scheme of present study

Our analysis has two parts (the analytical scheme is shown in Fig. 1). First, to describe the GWG distribution among Chinese women, subjects (*n* = 13,717) with an optimal pregnancy outcome were identified. A subject with an optimal outcome in our study was defined as a pregnant woman without prenatal medical complications (such as GDM or PIH), giving live birth at term (gestational age between 37 and 42 weeks), and with infant birth weight between 2500 and 3999 g. Among those with an optimal outcome, analysis of variance (ANOVA) test was used to examine the central tendency and variability for GWG by different maternal characteristics, including maternal age, parity, year of education, smoking during pregnancy and pre-pregnancy BMI group. The Student- Newman Keuls (SNK) test was adopted to make multiple comparisons when group categories were greater than two. The percentile distributions (5th, 15th, 25th, 50th, 75th, 85th, and 95th) of GWG according to both the Asian and IOM pre-pregnancy BMI cut-off points were compared. Observations regarding percentile trends were made on a descriptive basis, and statistical tests for trend were not reported. The middle 70% (Pc 15th–85th) and 50% (Pc 25th–75th) of the observed GWG distribution was compared to the IOM ranges.

Second, to examine which BMI classification scheme is more suitable for Chinese women, all subjects with an optimal GWG determined by the IOM BMI cut-off points (*n* = 6438) and those with optimal GWG determined by the Asian BMI cut-off points (*n* = 6110) were selected. Pregnancy outcomes were compared between those two groups. The crude odds ratios (OR) and 95% confidence intervals (CI) were calculated. Multivariate logistic regression models were also adopted to calculate adjusted OR and 95%CI. Potential confounding variables included maternal age (< 25, 25–34, and ≥ 35 years, 25–34 as reference), parity (primiparous or multiparous), year of education (≤12 years or > 12 years) and smoking during pregnancy (yes or no). Subgroup analyses were conducted among subjects with a pre-pregnancy BMI in the 23–24.9 and 25–29.9 strata. Statistical significance was assessed at the 0.05 level (two-tailed test). All analyses were performed using the SAS software, version 9.2 (SAS Institute, Inc., Cary, NC).

## Results

A total of 16,780 qualified subjects were enrolled in our study. Of them, 603 (3.59%) mothers were diagnosed with PIH, and 176 (1.05%) were diagnosed with GDM; 1249 (7.44%) births were preterm, 1001 (5.97%) were low birthweight, 1030 (6.14%) were macrosomia, 1389 (8.28%) were SGA and 1984 (11.82%) were LGA.

Based on IOM pre-pregnancy BMI cut-off points, there were 2651 (15.80%), 12,272 (73.13%), 1575 (9.39%) and 282 (1.68%) women defined as underweight, normal weight, overweight and obese, respectively, and 6438 (38.37%) women had an optimal GWG. According to Asian BMI cut-off points, there were 2651 (15.80%), 9897 (58.98%), 2375 (14.15%) and 1857 (11.07%) women classified as underweight, normal weight, overweight and obese, respectively, and 6110 (36.41%) women had an optimal GWG.

### Weight gain during pregnancy

After excluding those with preterm, LBW, macrosomia, PIH or GDM, 13,717 births with an optimal pregnancy outcome were selected to describe the distribution of GWG. Of them, the mean maternal age was 27.3 ± 4.44 years, the mean infant birthweight was 3274.9 ± 780.24 g, the mean pre-pregnancy BMI was 21.3 ± 3.15 kg/m^2^, and the mean GWG was 14.4 ± 5.39 kg.

**Fig. 2:**
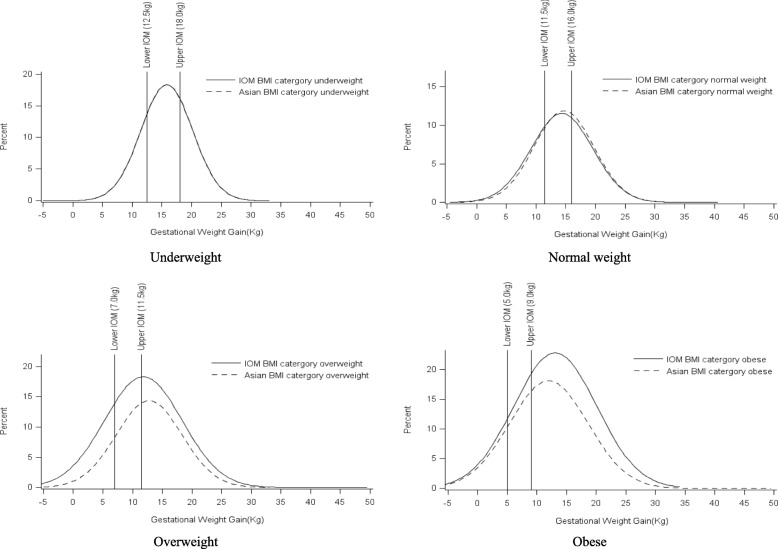
Comparison of gestational weight gain distribution by pre-pregnancy weight status to Institute of Medicine recommended ranges among Chinese with optimal pregnancy outcome. Vertical reference lines represent the lower and upper limit of the IOM gestational weight gain ranges for each pre-pregnancy body mass index (BMI) category

GWG was found to vary significantly by maternal age, parity, year of education, smoking during pregnancy, and both IOM and Asian pre-pregnancy BMI category (Table 2). Mothers who were 25–34 years old, primiparous, educated for less than 12 years, or smoked during pregnancy had a greater GWG than did the others. The relationship between GWG and pre-pregnancy BMI varied when different BMI classification schemes were used. Based on the Asian BMI classification scheme, the mean GWG decreased as pre-pregnancy BMI increased; the underweight group had the highest mean GWG, whereas the obese group had the lowest. However, when the IOM classification scheme was adopted, the relationship between GWG and pre-pregnancy BMI followed a U-shaped curve, and the overweight group had the lowest mean GWG (Table 2). The GWG percentile distribution analysis showed the same results (Table 3). Compared with the IOM BMI classification schemes, the shape and width of the GWG distributions resulting from the Asian BMI classification scheme were nearly identical for the underweight and normal weight groups, whereas curves for the overweight and obese groups differed in both height and the central position of the distribution (Table 3, Fig. 2).

**Table 2:** Gestational weight gain by maternal characteristics among subjects with optimal pregnancy outcome^c^ in Changsha, Hunan, China, in 2016

Characteristics	N (%)	Gestational weight gain				*p*
		Mean, kg	SD	Min/Max	CV, %	
Overall subjects	13,717 (100%)	14.3	5.31	−4.5/ 48.5	37.00	
Maternal age (years)						
≤ 24	3669 (25.75)	13.3^d^	5.74	−4.5/ 31.8	43.27	< 0.001
25–34	8724 (63.60)	14.8^d^	5.11	−4.5/ 48.5	34.58	
≥ 35	1324 (9.65)	14.5^d^	4.90	−3.0/ 34.0	33.87	
Parity						
Primiparous	9549 (69.61)	14.9	4.93	−4.5/ 48.5	32.97	< 0.001
Multiparous	4168 (30.39)	12.9	5.85	−4.5/ 31.5	45.17	
Education (years)						
≤ 12	8701 (64.37)	15.3	4.60	−4.5/ 32.5	21.19	< 0.001
≥ 13	5016 (36.57)	12.6	5.97	−4.5/ 48.5	47.29	
Smoking during pregnancy						
Yes	1461 (10.65)	15.7	4.27	−3.5/ 29.0	27.21	< 0.001
No	12,256 (89.35)	14.2	5.40	−4.5/ 48.5	38.05	
IOM pre-pregnancy BMI category ^a^						
Underweight	2261 (16.48)	15.8^d^	4.36	−4.5/ 31.5	27.54	< 0.001
Normal weight	10,114 (73.73)	14.3^d^	5.19	−4.5/ 40.0	36.20	
Overweight	1149 (8.38)	11.8^d^	6.54	−4.5/ 48.5	55.52	
Obese	193 (1.41)	13.1^d^	7.01	−4.0/ 29.5	53.66	
Asian pre-pregnancy BMI category ^b^						
Underweight	2261 (16.48)	15.8^d^	4.36	−4.5/ 31.5	27.54	< 0.001
Normal weight	8273 (60.31)	14.7^d^	5.04	−4.0/ 40.0	34.29	
Overweight	1841 (13.42)	12.7^d^	5.56	−4.5/ 32.5	43.62	
Obese	1342 (9.78)	12.0^d^	6.62	−4.5/ 48.5	55.35	

^a^IOM BMI category: underweight (BMI < 18.5), normal weight (BMI 18.5–24.9), overweight (BMI 25–29.9), obese (BMI ≥ 30)

^b^Asian BMI category: underweight (BMI < 18.5), normal weight (BMI 18.5–22.9), overweight (BMI 23–24.9), obese (BMI ≥ 25)

^c^Optimal outcome was defined as woman who has no prenatal medical complications (such as GDM or PIH), giving live birth at a gestational age between37 and 42 weeks, and infant birth weight between 2500 and 3999 g

^d^SNK test showed significant differences between each other, *p* < 0.05

**Table 3:** Distribution of gestational weight gain by pre-pregnancy BMI among subjects with optimal pregnancy outcomes in Changsha, Hunan, China, in 2016

Subgroups	Gestational weight gain (kg) by percentile							IOM recommend ranges (kg)
	5th	15th	25th	50th	75th	85th	95th	
Overall subjects (*n* = 12,564)	5.0	9.0	11.0	15.0	18.0	19.0	22.5	
IOM pre-pregnancy BMI category ^a^								
Underweight (*n* = 2002)	9.0	11.5	13.0	16.0	18.5	20.0	23.0	12.5–18.0
Normal weight (*n* = 9308)	5.0	9.0	11.0	15.0	17.5	19.0	22.5	11.5–16.0
Overweight (*n* = 1084)	0.0	4.5	7.5	12.5	16.0	18.0	21.5	7.0–11.5
Obese (n = 170)	2.5	5.0	8.0	13.0	17.5	20.0	26.0	5.0–9.0
Asian pre-pregnancy BMI category ^b^								
Underweight (n = 2002)	9.0	11.5	13.0	16.0	18.5	20.0	23.0	12.5–18.0
Normal weight (*n* = 7564)	6.0	10.0	11.6	15.0	18.0	19.0	22.8	11.5–16.0
Overweight (*n* = 1744)	3.0	7.0	9.5	13.0	16.5	18.0	21.0	7.0–11.5
Obese (*n* = 1254)	0.4	5.0	7.5	12.5	16.0	18.0	22.5	5.0–9.0

^a^: IOM pre-pregnancy BMI category: underweight (BMI < 18.5), normal weight (BMI 18.5–24.9), overweight (BMI 25–29.9), obese (BMI ≥ 30);

^b^: Asian pre-pregnancy BMI category: underweight (BMI < 18.5), normal weight (BMI 18.5–22.9), overweight (BMI 23–24.9), obese (BMI ≥ 25)

Note: Shaded areas represent the 2009 IOM gestational weight gain ranges

As shown in Table 3 and Fig. 2, the IOM recommendations by pre-pregnancy weight status are very narrow compared to the GWG distributions observed for Chinese women. In the both BMI classification schemes, the IOM recommended ranges are within the middle 70 and 50% of the observed distributions. Among underweight and normal weight women, the recommended IOM GWG ranges fall near the middle of the distribution. However, among overweight and obese women, the IOM recommended GWG ranges fall within the lower half of the distribution (Fig. 2).

### Adverse pregnancy outcomes and optimal GWG by different BMI classification

Compared with those with optimal GWG determined by IOM BMI cut-off points, women with optimal GWG determined by the Asian BMI cut-off points had lower risks of having macrosomia (crude OR = 0.79, 95%CI: 0.67–0.94) and LGA infant (crude OR = 0.86, 95%CI: 0.76, 0.97) (Table 4). After adjustments for maternal age, education, parity and smoking status during pregnancy, the associations still significantly exist. No significantly different risks of having preterm, LBW or SGA infants or different risks of having PIH or GDM were found between women with an optimal GWG determined by different BMI cut-off points (Table 4). The subgroup analysis in the pre-pregnancy BMI 23.0–24.9 and 25–29.9 strata showed the same results (Additional file 1: Table S1 and Additional file 2: Table S2).

**Table 4:** Comparison of adverse pregnancy outcomes between subjects with optimal GWG determined by the Asian BMI cut-offs^#^ and those with optimal GWG determined by the IOM BMI cut-offs^#^

Outcomes	Subjects with optimal GWG according to Asian BMI category^#^		Subjects with optimal GWG according to IOM BMI category^#^		Crude OR (95%CI)	Adjusted OR (95%CI)^a^
	N	%	N	%		
Preterm						
Yes	434	7.10	450	6.99	1.02(0.89, 1.17)	1.03(0.90, 1.19)
No	5676		5988		Reference	
Birth weight						
LBW	351	5.74	370	5.75	0.99(0.85, 1.15)	1.01(0.87, 1.17)
Macrosomia	242	3.96	319	4.95	0.79(0.67, 094)	0.79(0.67, 0.94)
Normal	5517		5749			
Birth weight by gestational age						
SGA	520	8.51	530	8.23	1.02(0.90, 1.16)	1.02(0.90, 1.16)
LGA	530	8.67	641	9.96	0.86(0.76, 0.97)	0.86(0.76, 0.98)
Normal	5060		5267			
PIH						
Yes	165	2.70	190	2.95	0.91(0.74, 1.13)	0.93(0.75, 1.15)
No	5945		6248			
GDM						
Yes	57	0.93	57	0.89	1.05(0.73, 1.52)	1.06(0.73, 1.53)
No	6053		6381			

^a^Adjustment covariates are maternal age, parity, education and smoking during pregnancy

^#^Optimal gestational weight gain determined by pre-pregnancy BMI category is listed in Table 1

## Discussion

Our data found that the GWG distribution for Chinese women varied with the use of different BMI classification schemes. The recommended IOM weight gain ranges are narrower than and shifted to the left of the actual distributions of GWG for Chinese women. We support the use of the Asian BMI categories to recommend and monitor target weight gains for Chinese pregnancies since lower risks of macrosomia and LGA were observed among those with an optimal GWG determined by the Asian BMI cut-off points.

### Weight gain during pregnancy among Chinese women

The GWG guidelines issued by the IOM are intended for use among American women but not for other women who are substantially shorter or thinner than American women [5, 18–20]. Studies validating these guidelines among Chinese women are emerging and have reached inconclusive results [9–13]. Yang et al. [9], using the IOM-recommended BMI cut-off points and combining the overweight and obese groups, concluded that IOM GWG recommendations are suitable for Chinese women. Jiang et al.[12] also used IOM BMI cut-off points but concluded, however, that the IOM-recommended GWG range is too high for the Chinese population pregnant with singletons. Zhou et al.[10] and Wong et al. [11], using Asian BMI cut-off points, found that the IOM GWG recommendations are narrower than observed among Chinese women. Yang S [13] enrolled 76,854 women in Wuhan and compared the ORs for abnormal birth weight and abnormal GWG determined using the IOM-recommended ranges and abnormal GWG determined using the quartile ranges observed from the study sample. He concluded that a GWG above the IOM recommendations might not be helpful for Chinese women. Our data support his finding that the IOM recommendations are narrower than the observed distribution and that the upper limits of the distribution are too restrictive for Chinese women. However, the IOM-recommended GWG ranges are still within the middle 70% of Chinese GWG distributions in both BMI classification schemes. Elevating the upper limit of the GWG ranges may lead to long-term adverse outcomes, including postpartum weight retention and future overweight and obesity in mother or offspring. Neither the previous studies [9–13] nor our study include data on postpartum outcomes. Therefore, whether Chinese women could gain more above the upper end of the IOM guidelines needs further investigation.

Previous researchers found that the GWG distribution among specific populations would vary by different pre-pregnancy BMI classification schemes [21, 22]. Our study found that this distribution variation also exists among Chinese women. This is consistent with previous findings [10, 11] that, based on the Asian BMI classification, found that the mean GWG among Chinese women decreased as pre-pregnancy BMI increased. However, based on the IOM BMI classification, we found that the relationship between GWG and pre-pregnancy BMI followed a U-shaped curve, which is inconsistent with Jiang’s result [12]. The sample sizes of women with a pre-pregnancy BMI greater than 30 strata in our study (*n* = 170) and Jiang’s study (*n* = 58) are both small. The reason for this inconsistency needs further research.

### BMI classification for GWG monitoring among Chinese women

Before 2018, no universal BMI cut-off points were recommended in China, and there were potential differences in the care of different BMI groups at the participating hospitals. The recommended weight gain ranges during pregnancy would vary when using different pre-pregnancy BMI cut-off points [5]. Our study found that women with an optimal GWG determined by the Asian BMI cut-off points had lower risks of macrosomia (OR = 0.78, 95%CI: 0.67, 0.94) and LGA (OR = 0.86, 95%CI: 0.76, 0.98) than women with an optimal GWG determined by IOM BMI cut-off points suggests that the Asian BMI cut-off points are more appropriate for Chinese women. In 2004, WHO experts declared that Asian populations have different associations between BMI and health risks than Western populations do [23]. Recommending GWG ranges based on the IOM BMI cut-off points would classify some Chinese women in lower pre-pregnancy BMI categories and suggest them gain more weight than necessary, which may put them at an unnecessarily higher risk for macrosomia and LGA [3]. In our sample, 13.42% of the women were misclassified as normal weight, and 8.37% were misclassified as overweight if the pre-pregnancy weight status was determined by the IOM BMI cut-off points instead of the Asian cut-off points. In Jan 2018, the national GWG guideline for Chinese women was published [8], and a universal recommendation with the IOM criteria may result in more misclassification and unnecessary weight gain among pregnant Chinese women in the future.

### Strengths and limitations

Strengths and limitations should be considered when interpreting the study findings. Information on birth weight and maternal complications was obtained from medical records, which minimized the potential misclassification of the outcomes. Information on gestational age and infant gender was available, which allowed us to not only control for gestational age when studying the relationship with LBW and macrosomia but also examine the association with SGA and LGA. Furthermore, the data contained detailed information on maternal demographic information, which allowed adjusting for several important potential confounding factors simultaneously. However, the limitation of our study should be considered when interpreting the study findings. Although weight at delivery was measured at the hospital and extracted from discharge abstracts, pre-pregnancy weight was recorded based on self-reported information. There is a potential misclassification for pre-pregnancy BMI status. However, high correlations were found between self-report and measured pre-pregnancy weight [24]. Our data are not from a nationally representative sample, we only used a retrospective cohort study in Changsha, which is in the central region of China, to eliminate the potential for selection bias. The generalizability of our findings and the appropriateness of the GWG guidelines by the Chinese Society of Gynecology and Obstetrics need further investigation.

## Conclusion

The IOM-recommended GWG ranges are within the middle 70% of the distributions in Chinese women. Pre-pregnancy weight status should be determined using the Asian BMI cut-off points when applying IOM GWG recommendations to Chinese women.

## Additional files

Additional files 1: **Table S1.** Subgroup analysis of comparison of adverse pregnancy outcomes between different gestational weight gain groups in the pre-pregnancy BMI 23.0–24.9 strata. (DOC 47 kb)

Additional files 2: **Table S2.** Subgroup analysis of comparison of adverse pregnancy outcome between different gestational weight gain groups in the pre-pregnancy BMI 25–29.9 strata. (DOC 47 kb)

## Abbreviations

BMI: Body mass index
CI: Confidence intervals
DBP: Diastolic blood pressure
GDM: Gestational diabetes mellitus
GWG: Gestational weight gain
IOM: Institute of Medicine
LGA: Large-for-gestational age
OR: Odds ratio
PIH: Pregnancy-induced hypertension
SBP: Systolic blood pressure
SGA: Small-for-gestational age
