# Supplementary material for: Gestational weight gain in Chinese women -- results from a retrospective cohort in Changsha, China
Source: BMC Pregnancy Childbirth. 2018 May 29;18:185. doi: 10.1186/s12884-018-1833-y (PMC5975263; doi:10.1186/s12884-018-1833-y)
Supplement: Supplementary file 1 — Table S1. Subgroup analysis of comparison of adverse pregnancy outcomes between different gestational weight gain groups in the pre-pregnancy BMI 23.0–24.9 strata. (DOC 47 kb) [file 12884_2018_1833_MOESM1_ESM.doc]

Table S1. Subgroup analysis of comparison of adverse pregnancy outcomes between different gestational weight gain groups in pre-pregnancy BMI 23.0- 24.9 strata

| Outcomes | | GWG  7- 11.5kg^a^ | | GWG  11.5-16 kg^b^ | | Crude OR (95%CI) | Adjusted OR (95%CI)^*^ |
| --- | --- | --- | --- | --- | --- | --- | --- |
|  |  | N | % | N | % |  |  |
| Preterm | |  |  |  |  |  |  |
|  | Yes | 65 | 10.25 | 63 | 7.39 | 1.43(0.99, 2.06) | 1.48(0.99, 2.11) |
|  | No | 569 |  | 789 |  | Reference |  |
| Birth weight | | |  |  |  |  |  |
|  | LBW | 52 | 8.20 | 51 | 5.99 | 1.30(0.87, 1.95) | 1.38(1.00, 1.97) |
|  | Macrosomia | 23 | 3.63 | 86 | 10.09 | 0.34(0.21, 0.55) | 0.31(0.19, 0.50) |
|  | Normal | 559 |  | 715 |  |  |  |
| Birth weight by gestational age | | | | | |  |  |
|  | SGA | 52 | 8.20 | 53 | 6.22 | 1.22(0.82, 1.83) | 1.28(0.85, 1.92) |
|  | LGA | 61 | 9.62 | 147 | 17.25 | 0.52(0.38, 0.72) | 0.51(0.36, 0.70) |
|  | Normal | 521 |  | 652 |  | Reference |  |
| PIH | |  |  |  |  |  |  |
|  | Yes | 23 | 3.63 | 39 | 4.58 | 0.79(0.46, 1.33) | 0.97(0.57, 1.66) |
|  | No | 611 |  | 813 |  | Reference |  |
| GDM | |  |  |  |  |  |  |
|  | Yes | 7 | 1.10 | 12 | 1.41 | 0.78(0.31, 1.99) | 0.79(0.30, 2.09) |
|  | No | 627 |  | 840 |  | Reference |  |

a According to Asian BMI category, women with pre-pregnancy BMI 23- 24.9 are classed as overweight whose recommended GWG range are 7- 11.5kg;

b According to IOM BMI category, women with pre-pregnancy BMI 23- 24.9 are classed as normal weight whose recommended GWG range are 11.5- 16 kg;

* Adjustment covariates are maternal age, parity, education and smoking during pregnancy.
