# Supplementary material for: Gestational weight gain in Chinese women -- results from a retrospective cohort in Changsha, China
Source: BMC Pregnancy Childbirth. 2018 May 29;18:185. doi: 10.1186/s12884-018-1833-y (PMC5975263; doi:10.1186/s12884-018-1833-y)
Supplement: Supplementary file 2 — Table S2. Subgroup analysis of comparison of adverse pregnancy outcome between different gestational weight gain groups in the pre-pregnancy BMI 25–29.9 strata. (DOC 47 kb) [file 12884_2018_1833_MOESM2_ESM.doc]

Table S2. Subgroup analysis of comparison of adverse pregnancy outcomes between different gestational weight gain groups in pre-pregnancy BMI 25- 29.9 strata

| Outcomes | | GWG  5- 9 kg^a^)= | | GWG  7- 11.5 kg^b^ | | Crude OR (95%CI) | Adjusted OR (95%CI)^*^ |
| --- | --- | --- | --- | --- | --- | --- | --- |
|  |  | N | % | N | % |  |  |
| Preterm | |  |  |  |  |  |  |
|  | Yes | 37 | 13.21 | 55 | 14.10 | 0.93(0.59, 1.45) | 1.06(0.66, 1.69) |
|  | No | 243 |  | 335 |  | Reference |  |
| Birth weight | | |  |  |  |  |  |
|  | LBW | 30 | 10.71 | 50 | 12.82 | 0.80(0.49, 1.30) | 0.91(0.55, 1.50) |
|  | Macrosomia | 22 | 7.86 | 36 | 9.23 | 0.82(0.47, 1.42) | 0.77(0.44, 1.36) |
|  | Normal | 228 |  | 304 |  |  |  |
| Birth weight by gestational age | | | | | |  |  |
|  | SGA | 25 | 8.93 | 34 | 8.72 | 1.00(0.58, 1.72) | 1.01(0.58, 1.75) |
|  | LGA | 43 | 15.36 | 68 | 17.44 | 0.86(0.56, 1.31) | 0.81(0.53, 1.24) |
|  | Normal | 212 |  | 288 |  | Reference |  |
| PIH | |  |  |  |  |  |  |
|  | Yes | 13 | 4.64 | 22 | 5.64 | 0.81(0.40, 1.65) | 0.88(0.43, 1.79) |
|  | No | 267 |  | 368 |  | Reference |  |
| GDM | |  |  |  |  |  |  |
|  | Yes | 15 | 5.36 | 10 | 2.56 | 2.15(0.95, 4.86) | 2.19(0.97, 4.99) |
|  | No | 265 |  | 380 |  | Reference |  |

a According to Asian BMI category, women with pre-pregnancy BMI 25-29.9 are classed as obese whose recommended GWG range are 5- 9 kg;

b According to IOM BMI category, women with pre-pregnancy BMI 25-29.9 are classed as overweight whose recommended GWG range are 7- 11.5 kg;

* Adjustment covariates are maternal age, parity, education and smoking during pregnancy.
